# Pharmacoeconomic evaluation of ICS-LABA therapy in pediatric asthma: a cost-effectiveness and cost-utility analysis

**DOI:** 10.3389/fphar.2025.1639444

**Published:** 2025-10-01

**Authors:** Fan Dai, Jin Xu, Jing Xu, Wenjing Li

**Affiliations:** Department of Pharmacy, Children’s Hospital of Nanjing Medical University, Nanjing, China

**Keywords:** pediatric asthma, ICS-LABA therapy, cost-effectiveness, cost-utility analysis, pharmacoeconomics, inhaled corticosteroids

## Abstract

**Background:**

Asthma is a chronic condition affecting children worldwide, with inhaled corticosteroid (ICS) and long-acting beta-agonist (LABA) combination therapies widely used in its management. However, the economic feasibility of these treatment regimens in pediatric asthma, particularly from a cost-effectiveness and cost-utility perspective, remains understudied. This retrospective study aimed to evaluate the clinical efficacy, cost-effectiveness, and cost-utility of multiple ICS-LABA therapy compared to single-inhaler therapy in children aged 0–18 years with asthma.

**Methods:**

A total of 59 pediatric patients diagnosed with asthma were included, divided into two main groups: the single-inhaler therapy group (Group A) and the multiple-inhaler regimens group (Group B). Group A consisted of subgroups A1 (salmeterol-fluticasone) and A2 (budesonide-formoterol), while Group B included subgroups B1 (two inhaled medications) and B2 (three inhaled medications). Clinical efficacy was measured based on symptom-free periods, while pharmacoeconomic analysis was conducted from the perspective of direct medical costs, including medication and non-medication costs. Both cost-effectiveness analysis (CEA) and cost-utility analysis (CUA) were employed, with outcomes presented as cost-effectiveness ratios (C/E), incremental cost-effectiveness ratios (ICER), and incremental cost-utility ratios (ICUR), using the willingness-to-pay (WTP) threshold of three times the *per capita* GDP of China.

**Results:**

The study found that while the total medication costs for multiple-inhaler regimens were higher than for single-inhaler therapies, the long-term cost-effectiveness and cost-utility favored multiple-inhaler regimens, especially triple regimens (Group B2) in complex and extended treatment scenarios. Shorter-term, less severe conditions were more economically manageable with single-agent therapies, with budesonide-formoterol (A2) showing superior cost-effectiveness over salmeterol-fluticasone (A1). Cost-effectiveness ratios (C/E) and incremental cost-effectiveness ratios (ICER) supported these findings. The cost-utility analysis, using QALYs, confirmed that multiple inhaler regimens were more cost-effective in children requiring prolonged treatment.

**Conclusion:**

This study provides important pharmacoeconomic insights into the treatment of pediatric asthma, highlighting the trade-offs between treatment costs and clinical outcomes. For children with more severe asthma, multiple ICS-LABA therapies, particularly triple therapy, offer better cost-effectiveness in the long term, while single-inhaler therapy remains economically viable for milder conditions. These findings support a stratified, individualized treatment approach and provide evidence for optimizing healthcare resource allocation in pediatric asthma management.

## Highlights

• Multiple ICS-LABA therapies, particularly triple therapy, offer better cost-effectiveness in the long term for children with more severe asthma.
• Single-inhaler therapy remains economically viable for children with milder conditions.
• Pharmacoeconomic evaluation of multiple ICS-LABA therapy in children aged 0–18 years with asthma may provide evidence for optimizing healthcare resource allocation in pediatric asthma management.

## 1 Introduction

Asthma is the most prevalent chronic respiratory disease in children, triggered by a variety of factors including infections, environmental allergens, genetic predispositions, and immune dysfunctions (Global Initiative for Asthma, 2025). Epidemiological studies from urban regions in China have reported a marked rise in childhood asthma prevalence, with a threefold increase among children under 14 years of age between 1990 and 2010. By 2010, the cumulative prevalence had reached 3.02% (The National Cooperative Gro up on Childhood Asthma, 2013). Clinically, asthma is characterized by recurrent episodes of wheezing, coughing, shortness of breath, and chest tightness - symptoms that often worsen at night or in the early morning hours (Global Initiative for Asthma, 2025; Gaillard et al., 2021; Editorial Board et al., 2020). These manifestations can significantly impair children’s health, quality of life, and academic performance, while placing considerable financial strain on families and pressure on public healthcare systems (Asher et al., 2021; Asher and Pearce, 2014).

The primary objective of asthma management is to achieve long-term disease control while minimizing exacerbations and adverse drug reactions. Current pharmacological therapies are broadly categorized into reliever medications, controller medications, and add-on therapies. Controller medications include inhaled corticosteroids (ICS), leukotriene receptor antagonists (LTRA), and fixed-dose combinations of ICS with long-acting β_2_-agonists (LABA). Pediatric asthma treatment is stratified by age (<6 years and ≥6 years), with therapy tailored to symptom severity and periodically adjusted to maintain optimal disease control.

For children under 6 years of age, the combination of ICS and LTRA remains the cornerstone of long-term management. Common ICS agents in China include budesonide, beclomethasone dipropionate, and fluticasone propionate, while montelukast sodium is the predominant LTRA. In children aged 6 years and older, ICS-LABA combination therapy is typically recommended. Frequently used ICS-LABA combinations include salmeterol-fluticasone dry powder inhalers and budesonide-formoterol inhalation powders. Due to limited evidence on the safety and efficacy of ICS-LABA combinations in children under 4 years of age, the Global Initiative for Asthma (GINA) does not currently recommend their use in this population. Nevertheless, the AUSTRI study—a multicenter, randomized, double-blind, prospective trial—demonstrated that fluticasone-salmeterol therapy reduced the risk of severe asthma exacerbations by 21% compared to fluticasone monotherapy, with a 35% reduction observed among adolescents. Both treatments exhibited similar rates of serious asthma-related adverse events (Stempel et al., 2016). These findings highlight the necessity of further research into the safety and efficacy of ICS-LABA therapy, particularly fluticasone-salmeterol, in younger pediatric populations (<6 years).

Asthma management increasingly follows a symptom-driven approach based on the “Assess–Adjust–Monitor” therapeutic cycle. Given asthma’s chronic course, long-term pharmacotherapy places substantial psychological and financial burdens on families and healthcare systems alike. Therefore, pharmacoeconomic evaluation of asthma therapies in children is essential. Utilizing a Markov model, Buendía et al. found that low-dose budesonide-formoterol was more cost-effective than ICS monotherapy for managing mild asthma in a Colombian cohort (Buendía and Patiño, 2021). Similarly, Zhou et al. showed that for Chinese patients aged ≥12 years, budesonide-formoterol used for both maintenance and relief offered better cost-effectiveness than fluticasone-salmeterol combined with salbutamol (Zhou et al., 2024). However, most existing pharmacoeconomic studies focus on adult or adolescent populations (≥12 years), and data on cost-effectiveness in children under 12 years remain scarce.

This study aims to evaluate the clinical efficacy and economic value of ICS-LABA monotherapy *versus* ICS-LABA combined with ICS therapy in pediatric asthma patients aged 0–18 years from a societal perspective in China. The analysis includes direct cost comparison, cost-effectiveness analysis, and cost-utility analysis using decision tree modeling. The results are intended to provide evidence-based insights to inform clinical decision-making and guide pharmaceutical policy for pediatric asthma management.

## 2 Materials and methods

### 2.1 Study subjects

This retrospective study included pediatric patients hospitalized in the Department of Respiratory Medicine at our hospital between January and December 2023 who received ICS-LABA combination inhalation therapy for asthma. Complete medical records documenting the full course of treatment since initial asthma diagnosis—including both inpatient care and outpatient follow-up were collected. The study compared the clinical efficacy and pharmacoeconomic outcomes of ICS-LABA monotherapy (single-inhaler regimen) *versus* combination therapy using multiple ICS-containing inhalers.

ICS-LABA inhalers included salmeterol/fluticasone (SF; Seretide, GlaxoSmithKline, H20150323, 50 μg/100 μg per blister, 60 blisters/box) and budesonide/formoterol (BF; Symbicort, AstraZeneca AB, H20140459, 80 μg/4.5 μg per actuation, 60 actuations/device). ICS-only inhalers included fluticasone propionate (FP; Flixotide, Glaxo Wellcome SA, H20130190, 125 μg per actuation, 120 actuations/canister).

The study was approved by the Ethics Committee of the Children’s Hospital of Nanjing Medical University. Informed consent was waived due to the retrospective nature of the study.

### 2.2 Inclusion and exclusion criteria

Inclusion criteria: (1) Diagnosis of asthma according to the 2016 guidelines of the Chinese Pediatric Society Respiratory Group (The Subspecialty Group of Respiratory Diseases, 2016); (2) Age ≤18 years; (3) Received either salmeterol/fluticasone or budesonide/formoterol during hospitalization.

Exclusion criteria: (1) Did not meet the above inclusion criteria; (2) Received inhaler therapy only during hospitalization with no available outpatient follow-up data; (3) Incomplete clinical records.

### 2.3 Data collection

#### 2.3.1 General information

Basic demographic data, including age, sex, and date of birth, were recorded.

#### 2.3.2 Clinical information

Both inpatient and outpatient medical records were reviewed.

Inpatient data included medical history (e.g., allergic comorbidities), hospitalization duration, asthma-related symptoms, total hospitalization costs, and medication-specific expenses (chemical medicines, traditional Chinese medicine, and inhalers).

Outpatient data included visit dates, symptom records, medication usage, total outpatient expenses, and detailed medication costs (including chemical medicines, traditional Chinese medicine, and inhalers).

### 2.4 Study design

#### 2.4.1 Grouping by treatment regimen

Patients were categorized based on the types of inhalers used throughout their treatment course:

Group A (single-inhaler regimens): Patients who used only one type of ICS-LABA inhaler, including Group A1 (SF only) and Group A2 (BF only);

Group B (multiple-inhaler regimens): Patients who used two or three inhalers, including at least one ICS-LABA inhaler, including Group B1 (Two inhalers, SF + FP, SF + BF, or FP + BF) and Group B2 (Three inhalers, SF + BF + FP).

#### 2.4.2 Age stratification

Patients were classified into five age groups: <1 year, 1–3 years, 4–7 years, 8–14 years, and >14 years.

#### 2.4.3 Seasonal distribution

Seasons were defined as: spring (March–May), summer (June–August), autumn (September–November), and winter (December–February).

#### 2.4.4 Evaluation of treatment effectiveness

The primary clinical goal of asthma management is to achieve remission. Remission is typically defined as the absence of significant asthma symptoms and acute exacerbations for at least 12 consecutive months, stable lung function, and mutual recognition of disease control by both physician and patient (Menzies-Gow et al., 2020). In preschool-aged children, predictors of remission include decreased frequency and severity of exacerbations and a declining trend in symptom burden (To et al., 2020; Longo et al., 2022). Based on these criteria, we defined three health states: symptom-free (no exacerbation), acute exacerbation requiring outpatient care, and acute exacerbation requiring hospitalization. No deaths occurred in the study cohort. Treatment effectiveness was evaluated by recording the number of medical visits and the longest symptom-free period under each health state. These metrics were compared between Group A and Group B to assess clinical outcomes.

#### 2.4.5 Pharmacoeconomic evaluation

Only direct medical costs were considered, including both pharmaceutical and non-pharmaceutical expenses (QI et al., 2015). All costs were calculated based on the 2019 Jiangsu Provincial Medical Service Price List and the 2019 centralized medicine bidding catalog. Total medication cost = ICS cost (SF, BF, FP) + traditional Chinese medicine cost + other chemical medicine cost. Unit prices: SF = ¥132.26/box, BF = ¥155.10/device, FP = ¥63.50/canister.

From the healthcare system perspective, two pharmacoeconomic methods were applied: cost-effectiveness analysis (CEA) and cost-utility analysis (CUA), to comprehensively assess the economic value of different treatment strategies (Higgins and Harris, 2012).

In CEA, Clinical effectiveness was defined by the longest duration of symptom-free status: ≥3 months (E_3_), ≥6 months (E_6_), ≥12 months (E_12_), and ≥24 months (E_24_). Costs included average ICS cost (C_I_) and total medication cost (C_M_). The cost-effectiveness ratio (C/E = cost/effect) and incremental cost-effectiveness ratio (ICER = Δcost/Δeffect) were calculated for each group.

In CUA, A decision tree model was constructed using TreeAge Pro 2022, with a simulation time horizon of 18 years. Effectiveness was measured in quality-adjusted life years (QALYs), and costs were based on either average ICS cost or total medication cost. The incremental cost-utility ratio (ICUR) was compared to a willingness-to-pay (WTP) threshold of ¥287,100/QALY (3× the 2024 *per capita* GDP in China, ¥95,700), in accordance with WHO guidelines and the 2020 China Guidelines for Pharmacoeconomic Evaluations (Liu et al., 2020). Health utility values were derived from the SYGMA2 study by FitzGerald et al. (FitzGerald et al., 2020): Symptom-free = 0.867; Acute exacerbation with outpatient treatment = −0.1; Acute exacerbation requiring hospitalization = −0.22 (based on the GOAL study) (Briggs et al., 2006).

To assess model robustness, probabilistic sensitivity analysis (PSA) was performed using Monte Carlo simulation (n = 1,000 iterations). Cost parameters followed a Gamma distribution, and probability parameters followed a Beta distribution. Results were visualized using cost-effectiveness plane scatter plots and cost-effectiveness acceptability curves (CEACs).

### 2.5 Statistical analysis

Statistical analysis was conducted using SPSS version 26.0. Continuous variables with normal distribution were expressed as mean ± standard deviation (x̄ ± s) and compared using the independent samples t-test. Categorical or non-normally distributed variables were presented as counts and percentages [n (%)] and compared using the chi-square test. A two-tailed P-value <0.05 was considered statistically significant.

## 3 Results

### 3.1 General and clinical information

#### 3.1.1 Demographic and baseline characteristics

A total of 59 pediatric asthma patients who received ICS-LABA combination inhalation therapy between January and December 2023 were included in the analysis based on the predefined inclusion and exclusion criteria. Of these, 30 patients were assigned to Group A (single-inhaler therapy: SF or BF), including 8 in subgroup A1 and 22 in subgroup A2. The remaining 29 patients were assigned to Group B (multiple-inhaler regimens: dual or triple ICS-LABA combinations), comprising 21 in subgroup B1 and 8 in subgroup B2.

In terms of sex distribution, male patients accounted for a significantly higher proportion than females (72.88% vs. 27.12%, P < 0.05). No significant sex difference was observed in Group A. However, Group B showed a significantly higher proportion of male patients (89.66%) compared to females (10.34%) (P < 0.05). Subgroup analysis revealed no significant sex differences between A1 and A2, whereas both B1 and B2 subgroups had significantly more males than females (P < 0.05).

The age of patients ranged primarily from 4 to 14 years. The mean age was 8.5 ± 2.1 years in Group A and 8.1 ± 2.1 years in Group B. Overall, children aged 4–7 years constituted the largest proportion (66.10%), followed by those aged 8–14 years (22.03%) and 1–3 years (10.17%). Only one patient was over 14 years old (in A2), and no children under 1 year were included. The age distribution in Group A was consistent with that of the overall cohort. In Group B, children aged 4–7 years remained predominant (79.31%), while the proportions of 1–3 and 8–14-year-olds were equal (10.34%). The mean ages in subgroups A1 and A2 were 6.9 ± 0.6 years and 9.1 ± 2.2 years, respectively, with A2 patients being significantly older than those in A1 (P < 0.05). Subgroup A1 consisted only of patients aged 1–3 years (12.50%) and 4–7 years (87.50%), while A2 included the largest proportion of patients aged 8–14 years (45.45%), followed by 4–7 years (40.91%), 1–3 years (9.09%), and >14 years (4.55%). The mean ages of patients in subgroups B1 and B2 were 8.0 ± 2.4 years and 8.6 ± 1.1 years, respectively, with no statistically significant difference between them. The age distribution in B1 was similar to that of the overall population, while B2 resembled the pattern seen in A1. Detailed baseline characteristics of the 59 pediatric patients are presented in Table 1.

**TABLE 1:** Baseline characteristics of 59 pediatric asthma patients, n (%).

Characteristic	A				B				Total	P
	A1	A2	Total	P	B1	B2	Total	P		
Total, n (%)	8 (26.67)	22 (73.33)	30 (50.85)	0.035*	21 (72.41)	8 (27.59)	29 (49.15)	0.046*	59 (100.0)	0.915
Sex, n (%)										
Male	5 (62.50)	12 (54.55)	17 (56.67)	0.840	18 (85.71)	8 (100.0)	26 (89.66)	0.795	43 (72.88)	0.258
Female	3 (37.50)	10 (45.45)	13 (43.33)	0.804	3 (14.29)	0 (0)	3 (10.34)	0.294	16 (27.12)	0.029*
P	0.562	0.728	0.551	-	0.006*	0.014*	<0.001*	-	0.004*	-
Age (Mean ± SD, years)	6.88 ± 0.64	9.09 ± 2.18	8.50 ± 2.13	0.003*	7.95 ± 2.38	8.63 ± 1.06	8.14 ± 2.10	0.072	8.32 ± 2.10	0.579
Age, n (%)										
<1 year	0 (0)	0 (0)	0 (0)	-	0 (0)	0 (0)	0 (0)	-	0 (100.0)	-
1–3 years	1 (12.50)	2 (9.09)	3 (10.0)	0.805	2 (9.52)	1 (12.50)	3 (10.34)	0.833	6 (10.17)	0.968
4–7 years	7 (87.50)	9 (40.91)	16 (53.33)	0.239	16 (76.19)	7 (87.50)	23 (79.31)	0.822	39 (66.10)	0.340
8–14 years	0 (0)	10 (45.45)	10 (33.33)	0.068	3 (14.29)	0 (0)	3 (10.34)	0.294	13 (22.03)	0.087
>14 years	0 (0)	1 (4.55)	1 (3.33)	0.549	0 (0)	0 (0)	0 (0)	-	1 (1.70)	0.329
P	0.005*	0.002*	<0.001*	-	<0.001*	0.005*	<0.001*	-	<0.001*	-

*P < 0.05.

#### 3.1.2 Clinical characteristics

Among the 59 pediatric patients, the total number of medical visits was significantly higher in Group B compared to Group A (P < 0.05). Subgroup analysis showed that patients in subgroup B2 had significantly more visits than those in B1, whereas no significant difference in visit frequency was observed between subgroups A1 and A2. No statistically significant difference was found in the length of hospitalization between Groups A and B or among their respective subgroups. However, the duration of outpatient follow-up was significantly longer in Group B than in Group A. With respect to seasonal distribution of asthma-related visits due to acute exacerbations, the number of visits during summer in subgroup A2 was significantly higher than in A1 (P < 0.05). No significant seasonal differences were observed among the other subgroups.

The majority of patients (86.44%) had a documented history of allergic conditions. There were no significant differences in the frequency of infections during hospitalization between Groups A and B or across the subgroups. A summary of the clinical characteristics of the 59 pediatric patients is provided in Table 2.

**TABLE 2:** Comparison of clinical characteristics of 59 pediatric asthma patients (Mean ± SD).

Characteristic	A				B				Total	P
	A1	A2	Total	P	B1	B2	Total	P		
Number of visits (times)	5.8 ± 3.8	8.6 ± 9.2	7.8 ± 8.2	0.887	16.4 ± 15.5	25.6 ± 13.1	18.9 ± 15.2	0.022*	13.3 ± 13.3	<0.001*
Duration of outpatient follow-up (months)	16.3 ± 16.1	20.5 ± 26.7	19.4 ± 24.1	0.677	35.4 ± 24.9	50.3 ± 17.81	39.5 ± 23.8	0.137	29.3 ± 25.9	0.002*
Length of hospitalization (days)	7.5 ± 2.0	8.2 ± 6.0	8.0 ± 5.3	0.759	7.0 ± 2.5	7.0 ± 2.7	7.0 ± 2.5	0.965	7.5 ± 4.1	0.341
Infections during hospitalization (times)	1.0 ± 0.0	1.1 ± 0.8	1.0 ± 0.7	0.873	1.0 ± 0.3	0.9 ± 0.4	1.0 ± 0.3	0.365	1.0 ± 0.5	0.624
Visits for acute asthma attacks (times)										
Spring	0.8 ± 0.9	0.6 ± 1.3	0.6 ± 1.2	0.678	0.8 ± 1.1	1.9 ± 1.8	1.1 ± 1.4	0.065	0.9 ± 1.3	0.138
Summer	0.0 ± 0.0	0.9 ± 0.9	0.7 ± 0.8	<0.001*	0.9 ± 1.2	0.8 ± 0.7	0.9 ± 1.1	0.671	0.8 ± 1.0	0.436
Autumn	0.4 ± 0.5	0.7 ± 0.7	0.6 ± 0.7	0.207	0.7 ± 0.9	0.9 ± 1.5	0.7 ± 1.1	0.646	0.7 ± 0.9	0.698
Winter	0.9 ± 1.0	0.6 ± 1.8	0.7 ± 1.6	0.725	0.9 ± 1.1	0.6 ± 0.5	0.8 ± 1.0	0.512	0.8 ± 1.3	0.716
P	0.064	0.018*	0.450	-	0.929	0.340	0.781	-	0.955	-

*P < 0.05.

### 3.2 Evaluation of treatment effectiveness

#### 3.2.1 Number of medical visits

Among the 59 pediatric patients, Group B accounted for 69.86% of the total medical visits, significantly higher than Group A (30.14%). Within Group A, patients in subgroup A2 contributed to a significantly greater proportion of visits (80.51%) compared to those in A1 (19.49%). Similarly, within Group B, the number of visits in subgroup B1 (62.89%) was significantly higher than in B2 (37.11%) (P < 0.05). Regarding hospitalizations, Group A had a higher proportion of inpatient visits (15.25%) than Group B (5.48%). Specifically, the hospitalization rate in subgroup A2 (77.78%) was significantly higher than that in A1 (22.22%), and in B1 (73.33%) compared to B2 (26.67%) (P < 0.05). For outpatient follow-up visits, Group B showed a significantly higher proportion (94.52%) compared to Group A (84.75%). Within Group A, patients in A2 accounted for 81.0% of outpatient visits, significantly more than those in A1 (19.0%). Similarly, in Group B, B1 accounted for a higher proportion of visits (62.28%) compared to B2 (37.72%) (P < 0.05). Across all subgroups (A1, A2, B1, and B2), the number of visits during symptom-free periods was significantly higher than those associated with asthma exacerbations. However, there were no significant differences in the number of exacerbation-related visits between subgroups A1 and A2 or between B1 and B2. A detailed breakdown of medical visit data for all 59 patients is presented in Table 3.

**TABLE 3:** Number of medical visits among 59 pediatric asthma patients, n (%).

Variable	A				B				Total	P
	A1	A2	Total	P	B1	B2	Total	P		
Visits, n (%)										
Total	46 (19.49)	190 (80.51)	236 (30.14)	<0.001*	344 (62.89)	203 (37.11)	547 (69.86)	<0.001*	783 (100.0)	<0.001*
Hospital visits	8 (22.22)	28 (77.78)	36 (15.25)	<0.001*	22 (73.33)	8 (26.67)	30 (5.48)	<0.001*	66 (8.43)	<0.001*
Outpatient visits	38 (19.0)	162 (81.0)	200 (84.75)	<0.001*	322 (62.28)	195 (37.72)	517 (94.52)	<0.001*	717 (91.57)	
Visits for onset	8 (21.05)	34 (20.99)	42 (21.00)	0.993	47 (14.60)	25 (12.82)	72 (13.93)	0.572	114 (15.90)	0.020*
Visits for no-onset	30 (78.95)	128 (79.01)	158 (79.00)	-	275 (85.40)	170 (87.18)	445 (86.07)	-	603 (84.10)	
P	0.003*	<0.001*	<0.001*	-	<0.001*	<0.001*	<0.001*	-	<0.001*	-
P	<0.001*	<0.001*	<0.001*	-	<0.001*	<0.001*	<0.001*	-	<0.001*	-

*P < 0.05.

#### 3.2.2 Longest symptom-free duration

To assess treatment effectiveness, we recorded the number of patients whose longest symptom-free duration reached ≥3 months, ≥6 months, ≥12 months, and ≥24 months in each study group. Across all four-time thresholds, a significantly higher proportion of patients in Group B achieved longer symptom-free periods compared to those in Group A (P < 0.05). However, no statistically significant differences were observed between subgroups A1 and A2 or between B1 and B2 for any of the duration categories. The distribution of the longest symptom-free durations among the 59 pediatric asthma patients is summarized in Table 4.

**TABLE 4:** Distribution of the longest symptom-free duration among 59 pediatric asthma patients, n (%).

Longest symptom-free duration	A				B				Total	P
	A1	A2	Total	P	B1	B2	Total	P		
≥3 months	6 (75.00)	11 (50.00)	17 (56.67)	0.222	18 (85.71)	8 (100.00)	26 (89.66)	0.259	43 (72.88)	0.004*
≥6 months	5 (62.50)	8 (36.36)	13 (43.33)	0.201	16 (76.19)	7 (87.50)	23 (79.31)	0.502	36 (61.02)	0.005*
≥12 months	3 (37.50)	8 (36.36)	11 (36.67)	0.954	14 (66.67)	6 (75.00)	20 (68.97)	0.665	31 (52.54)	0.013*
≥24 months	1 (12.50)	3 (13.64)	4 (13.33)	0.935	6 (28.57)	5 (62.50)	11 (37.93)	0.092	15 (25.42)	0.030*

*P < 0.05.

### 3.3 Pharmacoeconomic evaluation

#### 3.3.1 Comparison of treatment costs

The mean total treatment cost was ¥14,669.69 ± 12,240.63 in Group A and ¥16,884.22 ± 6,920.93 in Group B. Although the average cost in Group B was higher than in Group A, the difference was not statistically significant. Among subgroups, Group B2 had the highest mean total cost, which was significantly greater than that of Group B1 (P < 0.05). Group A1 had the lowest average cost, while Group A2 was higher than A1, though this difference was not statistically significant.

##### 3.3.1.1 Inpatient treatment cost comparison

The mean inpatient treatment cost was ¥11,620.08 ± 11,233.74 in Group A and ¥8,644.31 ± 2,903.51 in Group B. Although Group A had higher inpatient costs than Group B, the difference was not statistically significant. Within subgroups, Group A2 showed the highest inpatient cost, exceeding that of Group A1 without statistical significance. In Group B, the inpatient costs of subgroups B1 and B2 were comparable. The trends for average daily inpatient costs and non-medication inpatient costs were consistent with the total inpatient cost patterns.

In terms of inpatient medication costs, no significant differences were found between Groups A and B, nor among subgroups A1, A2, B1, and B2. For average daily inpatient medication costs, Group B was higher than Group A. Specifically, A1 was higher than A2, and B2 was higher than B1; however, none of these differences reached statistical significance.

Regarding inhaled corticosteroid (ICS) expenses during hospitalization, the average ICS cost was ¥180.03 ± 120.13 in Group A and ¥144.86 ± 11.56 in Group B, with Group A slightly higher but without statistical significance. Within subgroups, ICS costs were higher in A2 than A1, although the difference was not significant. The difference between B1 and B2 was also not statistically significant. However, the average daily ICS cost was significantly higher in Group A2 compared to A1 (P < 0.05), while no significant difference was observed between B1 and B2.

In terms of traditional Chinese medicine (TCM) costs during hospitalization, expenditures were comparable between Groups A and B. Group A1 had higher TCM costs than A2, and Group B2 was higher than B1, although these differences were not statistically significant. For other inpatient medications, the average cost was ¥1,785.08 ± 1,748.73 in Group A and ¥1,609.27 ± 896.25 in Group B, with Group A being slightly higher. Again, no statistically significant differences were observed between the groups or among the subgroups. Daily costs for both TCM and other medications were generally higher in Group B compared to Group A, with A1 exceeding A2 and B2 exceeding B1, though none of these differences were statistically significant. Detailed cost comparisons are presented in Table 5.

**TABLE 5:** Comparison of inpatient treatment costs among groups (Mean ± SD, CNY).

Cost	A				B				P
	A1	A2	Total	P	B1	B2	Total	P	
Total treatment cost	10124.89 ± 3039.89	12163.79 ± 13039.56	11620.08 ± 11233.74	0.668	8639.80 ± 2651.26	8656.15 ± 3692.95	8644.31 ± 2903.51	0.989	0.172
Average daily treatment cost	1389.98 ± 465.94	1520.64 ± 704.99	1485.80 ± 644.80	0.888	1312.85 ± 414.67	1277.52 ± 368.60	1303.11 ± 396.29	0.922	0.404
Non-pharmacological cost	8135.29 ± 2839.74	10184.09 ± 11012.27	9637.74 ± 9519.03	0.611	6892.96 ± 2128.60	6816.33 ± 3033.16	6871.82 ± 2353.21	0.939	0.134
Daily non-pharmacological cost	112.59 ± 461.11	1291.66 ± 649.77	1246.57 ± 602.36	0.606	1064.64 ± 389.21	1024.17 ± 384.53	1053.47 ± 381.45	0.770	0.249
Total medication cost	1989.60 ± 977.39	1979.70 ± 2115.36	1982.34 ± 1863.04	0.990	1746.83 ± 892.26	1839.83 ± 910.64	1772.49 ± 881.91	0.805	0.585
Daily medication cost	267.40 ± 101.33	228.98 ± 99.67	239.23 ± 99.85	0.373	248.21 ± 84.65	253.35 ± 64.49	249.63 ± 78.51	0.884	0.544
ICS cost	132.26 ± 0.00	197.40 ± 136.91	180.03 ± 120.13	0.194	145.31 ± 11.58	143.68 ± 12.21	144.86 ± 11.56	0.741	0.121
Daily ICS cost	18.78 ± 4.99	28.42 ± 14.59	25.85 ± 13.37	0.038*	23.48 ± 8.99	23.68 ± 9.76	23.54 ± 9.03	0.883	0.831
TCM cost	20.72 ± 58.60	15.97 ± 51.02	17.24 ± 52.14	0.830	17.23 ± 39.81	21.32 ± 39.75	18.36 ± 39.12	0.806	0.926
Daily TCM cost	2.07 ± 5.86	0.89 ± 2.67	1.21 ± 3.70	0.968	2.95 ± 7.26	3.86 ± 7.42	3.20 ± 7.18	0.730	0.378
Other medication cost	1836.62 ± 990.70	1766.34 ± 1973.44	1785.08 ± 1748.73	0.924	1584.29 ± 904.82	1674.82 ± 931.19	1609.27 ± 896.25	0.813	0.631
Daily other medication cost	246.54 ± 103.69	199.67 ± 101.93	212.17 ± 102.78	0.260	221.78 ±	225.81 ± 74.65	222.90 ± 84.18	0.922	0.544

*P < 0.05.

##### 3.3.1.2 Outpatient treatment cost comparison

Overall, Group B incurred higher outpatient treatment costs than Group A during both asthma exacerbations and symptom-free periods. Notably, the difference during symptom-free visits was statistically significant (P < 0.05). Subgroup analysis revealed that during exacerbations, Group A2 had higher outpatient costs than A1, whereas during symptom-free periods, A1 had higher costs than A2. In Group B, B2 incurred higher costs than B1 in both clinical states; however, these differences were not statistically significant.

Overall, Group B incurred higher outpatient costs than Group A during both asthma exacerbation and symptom-free periods, with the difference during symptom-free visits being statistically significant (P < 0.05). Subgroup analysis showed that during asthma exacerbation, Group A2 had higher outpatient costs than A1, whereas the opposite was observed during symptom-free periods. Group B2 showed higher costs than B1 in both scenarios, but the differences were not statistically significant. Regarding monthly average outpatient costs, Group A had higher expenses in both states compared to Group B, with no significant differences. Within subgroups, A1 had higher monthly costs than A2 during exacerbations, while A2 had higher costs during symptom-free periods. In contrast, B1 showed higher monthly costs than B2 in both states, but again, without statistical significance. In terms of monthly average outpatient costs, Group A had higher expenditures than Group B during both exacerbations and symptom-free periods, though the differences were not statistically significant. Within Group A, A1 had higher monthly costs than A2 during exacerbations, while A2 had higher costs during symptom-free periods. In Group B, B1 showed higher monthly costs than B2 during exacerbations, whereas B2 exceeded B1 during symptom-free periods; again, none of these differences reached statistical significance.

For non-medication outpatient treatment costs during asthma exacerbations, no significant difference was observed between Groups A and B. However, Group A2 had higher costs than A1, and Group B2 had higher costs than B1, although these differences were not statistically significant. During symptom-free visits, Group B had significantly higher non-medication costs than Group A (P < 0.05). Subgroup analysis showed that A2 had higher costs than A1 (not significant), while B2 was significantly higher than B1 (P < 0.05). Monthly average non-medication costs were higher in Group A than in Group B, but this difference was not significant. Within Group A, A1 had higher costs than A2 during exacerbations, while A2 had higher costs during symptom-free periods. In Group B, B1 had higher costs than B2 in both clinical states, but again, differences were not statistically significant.

Regarding outpatient medication costs, Group B had significantly higher expenditures than Group A during both exacerbations and symptom-free periods (P < 0.05). Group A2 and B2 showed higher costs than A1 and B1, respectively, but the differences were not significant. For monthly average medication costs, Group A had higher values than Group B, though the difference was not statistically significant. A1 had significantly higher monthly medication costs than A2 during exacerbations (P < 0.05), while A2 had higher costs during symptom-free periods. In Group B, B1 had higher monthly costs than B2 during exacerbations, whereas B2 exceeded B1 during symptom-free periods; none of these subgroup differences reached statistical significance.

For ICS costs during outpatient visits, Group B had significantly higher expenses than Group A in both clinical states (P < 0.05). However, differences among subgroups were not statistically significant. Group A showed higher monthly average ICS costs, with Group A2 significantly higher than A1 during exacerbations (P < 0.05), and also higher during symptom-free periods (not significant). Within Group B, B1 had higher monthly costs than B2 during exacerbations, while B2 exceeded B1 during symptom-free periods; these differences were not statistically significant.

In terms of outpatient TCM costs, Group A had slightly higher expenses than Group B during exacerbations, though the difference was not statistically significant. Costs between A1 and A2 were similar, while B1 was marginally higher than B2. During symptom-free periods, Group B had significantly higher TCM costs than Group A (P < 0.05), though subgroup differences were not statistically significant. For monthly average TCM costs, Group A was higher during exacerbations, and Group B was higher during symptom-free periods, with neither difference reaching statistical significance. A1 had significantly higher monthly TCM costs than A2 during exacerbations (P < 0.05), while A2 had higher costs than A1 during symptom-free periods. B1 had higher costs than B2 in both clinical states, though not statistically significant.

As for other outpatient medication costs, Group B had significantly higher costs than Group A in both clinical states (P < 0.05). Subgroup comparisons showed that A2 and B2 had higher costs than A1 and B1, respectively, though these differences were not statistically significant. For monthly average costs, Group A was higher than Group B during exacerbations, whereas Group B was higher during symptom-free periods; neither difference was statistically significant. Within Group A, A2 had higher costs than A1 during exacerbations, while A1 exceeded A2 during symptom-free periods. Similarly, in Group B, B1 had higher costs than B2 during exacerbations, and B2 exceeded B1 during symptom-free periods; none of these comparisons reached statistical significance. Detailed cost data are presented in Table 6.

**TABLE 6:** Comparison of outpatient treatment costs among groups (Mean ± SD, CNY).

Cost	A				B				P
	A1	A2	Total	P	B1	B2	Total	P	
During asthma exacerbation									
Total cost	763.96 ± 626.31	1691.47 ± 1518.80	1364.11 ± 1331.56	0.478	1295.08 ± 858.40	2409.54 ± 2988.67	1666.57 ± 1867.36	0.225	0.134
Monthly average cost	240.44 ± 325.73	161.90 ± 216.88	189.62 ± 253.08	0.260	79.11 ± 137.04	49.24 ± 59.04	69.15 ± 116.26	0.812	0.254
Non-medication cost	521.27 ± 642.63	1117.77 ± 1107.85	907.24 ± 991.20	0.605	667.30 ± 536.32	1406.77 ± 1751.37	913.79 ± 1117.10	0.194	0.343
Monthly avg. non-medication cost	158.41 ± 242.29	116.90 ± 194.22	131.55 ± 205.76	0.056	51.36 ± 121.95	29.22 ± 35.12	43.98 ± 100.93	0.803	0.281
Medication cost	242.69 ± 142.37	573.70 ± 543.66	456.87 ± 466.53	0.615	627.79 ± 424.47	1002.77 ± 1263.44	752.78 ± 797.45	0.286	0.028*
Monthly avg. medication cost	82.03 ± 141.59	45.00 ± 44.28	58.07 ± 88.45	0.043*	27.75 ± 22.18	20.02 ± 24.48	25.17 ± 22.74	0.908	0.388
ICS cost	44.09 ± 68.30	141.00 ± 213.28	106.80 ± 179.35	0.441	159.81 ± 125.69	327.08 ± 285.20	215.56 ± 203.83	0.085	0.012*
Monthly avg. ICS cost	3.60 ± 7.57	12.70 ± 24.08	9.49 ± 20.01	0.029*	5.30 ± 5.27	6.97 ± 5.90	5.86 ± 5.42	0.197	0.863
TCM cost	84.59 ± 142.01	85.83 ± 132.06	85.39 ± 131.16	0.365	94.41 ± 147.26	18.66 ± 40.08	69.16 ± 126.34	0.113	0.763
Monthly avg. TCM cost	62.94 ± 145.86	2.12 ± 3.54	23.59 ± 86.91	0.048*	3.97 ± 6.90	0.39 ± 0.80	2.78 ± 5.85	0.070	0.370
Other meds cost	114.01 ± 156.17	346.87 ± 326.61	264.68 ± 295.72	0.414	373.57 ± 293.07	657.03 ± 964.31	468.06 ± 598.05	0.317	0.043*
Monthly avg. other meds cost	15.49 ± 25.65	30.17 ± 42.28	24.99 ± 37.09	0.787	18.47 ± 20.26	12.66 ± 18.64	16.54 ± 19.53	0.860	0.943
During symptom-free visits									
Total cost	2583.89 ± 3535.76	2267.95 ± 2602.01	2355.11 ± 2822.13	0.724	5567.06 ± 5906.68	10256.44 ± 5232.16	6860.68 ± 6026.13	0.060	0.001*
Monthly average cost	229.62 ± 235.38	324.58 ± 335.45	298.38 ± 309.99	0.539	252.84 ± 307.07	230.32 ± 188.90	246.63 ± 276.36	0.849	0.587
Non-medication cost	931.50 ± 862.73	1143.78 ± 1181.99	1085.22 ± 1092.39	0.716	2500.68 ± 2107.01	4332.94 ± 1583.35	3006.13 ± 2119.53	0.035*	<0.001*
Monthly avg. non-medication cost	83.09 ± 83.54	166.29 ± 178.48	143.34 ± 161.03	0.260	129.39 ± 202.92	92.35 ± 40.24	119.17 ± 173.49	0.616	0.657
Medication cost	1652.40 ± 2924.12	1124.17 ± 1546.75	1269.89 ± 1975.91	0.483	3066.38 ± 4076.71	5923.50 ± 5123.06	3854.55 ± 4220.19	0.104	0.004*
Monthly avg. medication cost	146.53 ± 187.56	158.29 ± 197.03	155.05 ± 191.19	0.955	123.45 ± 153.57	137.97 ± 152.16	127.46 ± 150.59	0.821	0.618
ICS cost	247.99 ± 148.92	502.23 ± 702.08	432.09 ± 609.10	0.360	857.25 ± 1337.13	1881.11 ± 1188.74	1139.70 ± 1359.14	0.069	0.012*
Monthly avg. ICS cost	44.36 ± 45.82	90.18 ± 93.71	77.54 ± 85.04	0.240	29.11 ± 27.50	35.48 ± 17.54	30.87 ± 25.01	0.550	0.010*
TCM cost	87.70 ± 100.91	188.41 ± 437.03	160.63 ± 375.59	0.556	422.80 ± 483.44	614.13 ± 466.06	475.58 ± 478.35	0.345	0.006*
Monthly avg. TCM cost	6.94 ± 9.35	15.33 ± 39.45	13.02 ± 33.89	0.585	18.79 ± 26.70	12.06 ± 5.81	16.94 ± 22.96	0.490	0.563
Other meds cost	1316.71 ± 2833.15	433.53 ± 602.72	677.17 ± 1558.06	0.400	1786.33 ± 3237.53	3428.26 ± 3982.17	2239.27 ± 3465.41	0.262	0.030*
Monthly avg. other meds cost	95.23 ± 187.16	52.78 ± 108.94	64.49 ± 132.69	0.416	75.54 ± 140.36	90.43 ± 144.06	79.65 ± 138.95	0.802	0.624

*P < 0.05.

#### 3.3.2 Pharmacoeconomic analysis

This study employed cost-effectiveness analysis (CEA) and cost-utility analysis (CUA) to evaluate both the total pharmacotherapy costs and those specifically associated with inhaled corticosteroid (ICS) therapy.

##### 3.3.2.1 Cost-effectiveness analysis (CEA)

The clinical improvement rate, defined as the proportion of patients achieving the longest symptom-free durations of ≥3 months, ≥6 months, ≥12 months, and ≥24 months, was used as the measure of treatment effectiveness—denoted as E_3_, E_6_, E_12_, and E_24_, respectively. The total medication cost (C_M_) and average ICS cost (C_I_) for each group were recorded to calculate cost-effectiveness ratios (C/E). Additionally, incremental cost-effectiveness ratios (ICERs) were computed to compare the economic efficiency between groups.

The total pharmacotherapy cost in Group A was significantly lower than in Group B (P < 0.05). However, only the C_M_/E_3_ value was lower in Group A; for E_6_, E_12_, and E_24_, Group A’s C/E values were all higher than those of Group B. Among the ICER values, only ΔC_I_/ΔE_3_ in Group B exceeded the corresponding C/E of Group A, while all other ICERs were lower. These findings indicate that Group B demonstrated better cost-effectiveness for achieving longer symptom-free durations, whereas Group A was more economically favorable for short-term outcomes.

Subgroup analysis revealed no statistically significant difference in total medication cost between Group A1 and Group A2, though A1 had a slightly higher average cost. Group A2 had lower C/E values than A1 for C_M_/E_12_ and C_M_/E_24_ only; for the other indicators, A2’s values were higher. ICER results showed that only ΔC_M_/ΔE_6_ and ΔC_M_/ΔE_12_ were lower than the corresponding C/E values in A2, whereas ΔC_M_/ΔE_3_ exceeded A2’s C/E, and ΔC_M_/ΔE_24_ was negative. These results suggest that Group A1 was more cost-effective than A2 in short-term treatment durations. In Group B, B1 had significantly lower total medication costs than B2 (P < 0.05). Group B1 exhibited lower C/E values than B2 for all durations except C_M_/E_24_. Furthermore, all ICERs (except for ΔC_M_/ΔE_24_) were higher than the corresponding C/E values in B1, indicating that Group B1 was more cost-effective overall. However, B2 showed some economic advantage in long-term therapy. From a total medication cost perspective, three inhalers involving ICS-LABA combined with additional medications (e.g., SF + BF + FP) were more cost-effective for patients requiring complex, long-term treatment, compared to two inhalers involving ICS-LABA combined with additional medications (e.g., SF + BF, SF + FP, or BF + FP). Conversely, for milder conditions or short-term therapy, single-inhaler ICS-LABA regimens proved more economically advantageous—among which, SF was more cost-effective than BF. Detailed CEA data for total medication costs are presented in Table 7.

**TABLE 7:** Cost-effectiveness analysis of total medication treatment (C_M_).

Variable	A				B				P
	A1	A2	Total	P	B1	B2	Total	P	
Average Medication cost (C_M_)	3824.02	3339.63	3468.8	0.425	5291.53	8766.09	6250.03	0.013*	<0.001*
Effectiveness rate (E, %)									
E_3_	75.00	50.00	56.67	0.222	85.71	100.00	89.66	0.259	0.004*
E_6_	62.50	36.36	43.33	0.201	76.19	87.50	79.31	0.502	0.005*
E_12_	37.50	36.36	36.67	0.954	66.67	75.00	68.97	0.665	0.013*
E_24_	12.50	13.64	13.33	0.935	28.57	62.50	37.93	0.092	0.030*
Cost- effectiveness rate (C_M_/E)									
C_M_/E_3_	50.99	66.79	61.21	-	61.74	87.66	69.71	-	-
C_M_/E_6_	61.18	91.85	80.06	-	69.45	100.18	78.81	-	-
C_M_/E_12_	101.97	91.85	94.60	-	79.37	116.88	90.62	-	-
C_M_/E_24_	305.92	244.84	260.23	-	185.21	140.26	164.78	-	-
Incremental cost- effectiveness rate (∆C_M_/∆E)									
∆C_M_/∆E_3_	19.38	-	-	-	-	243.15	84.31	-	-
∆C_M_/∆E_6_	18.53	-	-	-	-	307.21	77.30	-	-
∆C_M_/∆E_12_	424.90	-	-	-	-	417.11	86.11	-	-
∆C_M_/∆E_24_	−424.90	-	-	-	-	102.40	113.06	-	-

*P < 0.05.

With regard to ICS therapy costs, Group A exhibited lower C_I_/E_3_, C_I_/E_6_, and C_I_/E_12_ values compared to Group B, whereas the C_I_/E_24_ value was higher. ICS costs were significantly higher in Group B than in Group A (P < 0.05). Therefore, Group A, as the lower-cost group, was used as the reference in ICER calculations. The incremental cost-effectiveness ratios (∆C_I_/∆_E_) between Groups A and B were all greater than zero. Specifically, Group B’s ∆C_I_/∆E_3_, ∆C_I_/∆E_6_, and ∆C_I_/∆E_12_ values exceeded Group A’s corresponding C_I_/E values, while ∆C_I_/∆E_24_ was lower. These results suggest that Group B was generally not more economically favorable than Group A, though it demonstrated some cost-effectiveness advantage in long-term, complex treatment scenarios.

Subgroup analysis within Group A showed that all C_I_/E values in A1 were lower than those in A2. Given A1’s slightly lower ICS cost, ICERs calculated using A1 as the reference indicated that ∆C_I_/∆E_3_, ∆C_I_/∆E_6_, and ∆C_I_/∆E_12_ were negative, suggesting that A2 was a dominated strategy—i.e., it incurred higher costs with lower effectiveness. Although ∆C_I_/∆E_24_ was positive and exceeded A1’s C_I_/E_24_, A2 still lacked a cost-effectiveness advantage over A1, even in long-term treatment. In Group B, B1 demonstrated lower C_I_/E_3_, C_I_/E_6_, and C_I_/E_12_ values than B2, while C_I_/E_24_ was higher. As B2 had significantly higher ICS costs (P < 0.05), B1 was used as the reference for ICER calculations. The ∆C_I_/∆_E_ values between B1 and B2 were all positive, and B2’s ∆C_I_/∆E_3_, ∆C_I_/∆E_6_, and ∆C_I_/∆E_12_ were all higher than B1’s corresponding C/E values. In contrast, ∆C_I_/∆E_24_ was lower than B1’s C_I_/E_24_. These findings indicate that B2 was not cost-effective compared to B1, except possibly in long-term or more severe cases. In summary, based on ICS therapy cost analysis, single-inhaler therapy with a single ICS agent was more economically favorable for patients with mild or short-term disease, with SF being more cost-effective than BF. For more complex or long-term cases, two inhalers involving ICS-LABA (e.g., SF + BF, SF + FP, or BF + FP) demonstrated better cost-effectiveness than three inhalers involving ICS-LABA (SF + BF + FP). The detailed ICS cost-effectiveness results are presented in Table 8.

**TABLE 8:** Cost-effectiveness analysis of ICS treatment (C_I_).

Variable	A				B				P
	A1	A2	Total	P	B1	B2	Total	P	
Average ICS cost (C_I_)	413.31	747.30	658.24	0.600	1124.32	2351.86	1462.96	0.007*	0.001*
Effectiveness rate (E, %)									
E_3_	75.00	50.00	56.67	0.222	85.71	100.00	89.66	0.259	0.004*
E_6_	62.50	36.36	43.33	0.201	76.19	87.50	79.31	0.502	0.005*
E_12_	37.50	36.36	36.67	0.954	66.67	75.00	68.97	0.665	0.013*
E_24_	12.50	13.64	13.33	0.935	28.57	62.50	37.93	0.092	0.030*
Cost- effectiveness rate (C_I_/E)									
C_I_/E_3_	5.51	14.95	11.62	-	13.12	23.52	16.32	-	-
C_I_/E_6_	6.61	20.55	15.19	-	14.76	26.88	18.45	-	-
C_I_/E_12_	11.02	20.55	17.95	-	16.86	31.36	21.21	-	-
C_I_/E_24_	33.06	54.79	49.38	-	39.35	37.63	38.57	-	-
Incremental cost- effectiveness rate (∆C_I_/∆E)									
∆C_I_/∆E_3_	-	−13.36	-	-	-	85.90	24.39	-	-
∆C_I_/∆E_6_	-	−12.78	-	-	-	108.54	22.37	-	-
∆C_I_/∆E_12_	-	−292.97	-	-	-	147.36	24.91	-	-
∆C_I_/∆E_24_	-	292.97	-	-	-	36.18	32.71	-	-

*P < 0.05.

By integrating the cost-effectiveness results of both total pharmacotherapy and ICS-specific treatment, it can be concluded that in complex or long-duration cases, multiple-inhaler regimens are generally more cost-effective than single-inhaler therapy. Among these, dual ICS combinations offer greater economic advantages in terms of ICS-specific costs, whereas triple combinations may provide slight benefits in overall medicine expenditure. Conversely, for milder conditions or short-term treatment, single-inhaler therapy more economical, with salmeterol/fluticasone (SF) being more cost-effective than budesonide/formoterol (BF).

##### 3.3.2.2 Cost-utility analysis (CUA)

Cost-utility analysis was conducted using both total pharmacotherapy costs and ICS-specific costs as economic inputs, with utility measured in quality-adjusted life years (QALYs).

When total pharmacotherapy costs were considered, the mean total costs for Groups A and B were ¥1,233.82 and ¥3,332.05, respectively, while the corresponding utility values were 14.68 QALYs and 15.15 QALYs. The incremental cost-utility ratio (ICUR) for Group B *versus* Group A was ¥4,464.17/QALY, which is well below the willingness-to-pay (WTP) threshold of ¥287,100 (three times China’s *per capita* GDP). This indicates that the use of multiple ICS agents (SF, BF, FP) throughout the treatment course—encompassing both inpatient and outpatient care—is more cost-effective than single-inhaler therapy with SF or BF. In subgroup analysis, Group B2 incurred ¥2,507.55 more in total pharmacotherapy costs than Group B1 and achieved an additional 0.12 QALYs, resulting in an ICUR of ¥20,632.18/QALY—also well within the WTP threshold. This suggests that triple therapy is more cost-effective than dual therapy. Conversely, within the single-inhaler therapy groups, Group A1 (SF) had ¥314.09 lower costs than Group A2 (BF) but also yielded 0.1 fewer QALYs, resulting in an ICUR of -¥3,273.86/QALY. Despite being negative, this value still indicates that BF is more cost-effective than SF, as it provides higher utility at a modestly increased cost within acceptable economic limits.

When ICS-specific costs were analyzed, Group A and Group B had average ICS costs of ¥335.75 and ¥963.47, respectively, with identical QALY values as above. The ICUR for Group B *versus* Group A was ¥1,335.52/QALY, confirming the economic advantage of multiple ICS agents over single-agent therapy. Within the multiple-inhaler regimens groups, Group B2 incurred ¥904.78 more in ICS-related costs than Group B1 and gained 0.12 QALYs, again yielding an ICUR of ¥20,632.18/QALY. In the single-agent groups, Group A2 had ¥200.27 higher ICS costs than Group A1 and yielded an additional 0.1 QALYs, resulting in an ICUR of ¥2,087.43/QALY—well below the WTP threshold. These findings consistently demonstrate that BF is more cost-effective than SF in single-inhaler therapy.

**FIGURE 1:**
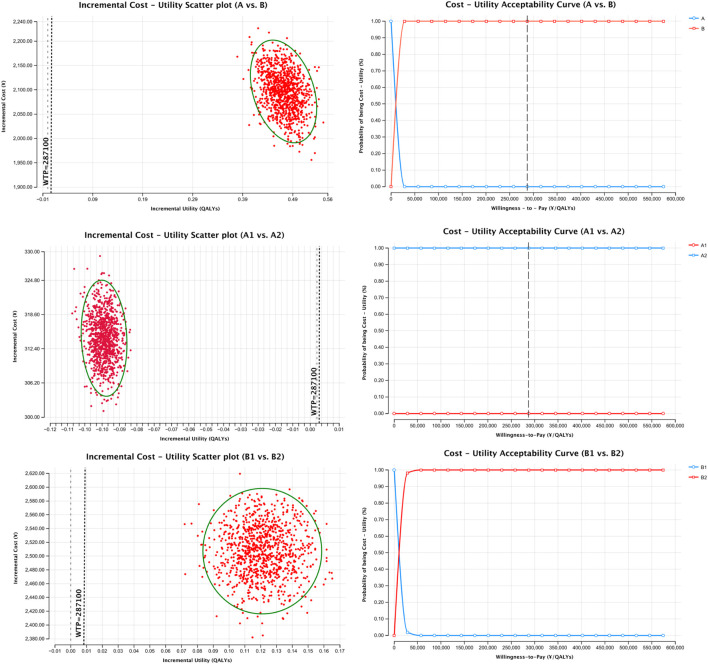
Sensitivity analysis of total medication cost-utility.

**FIGURE 2:**
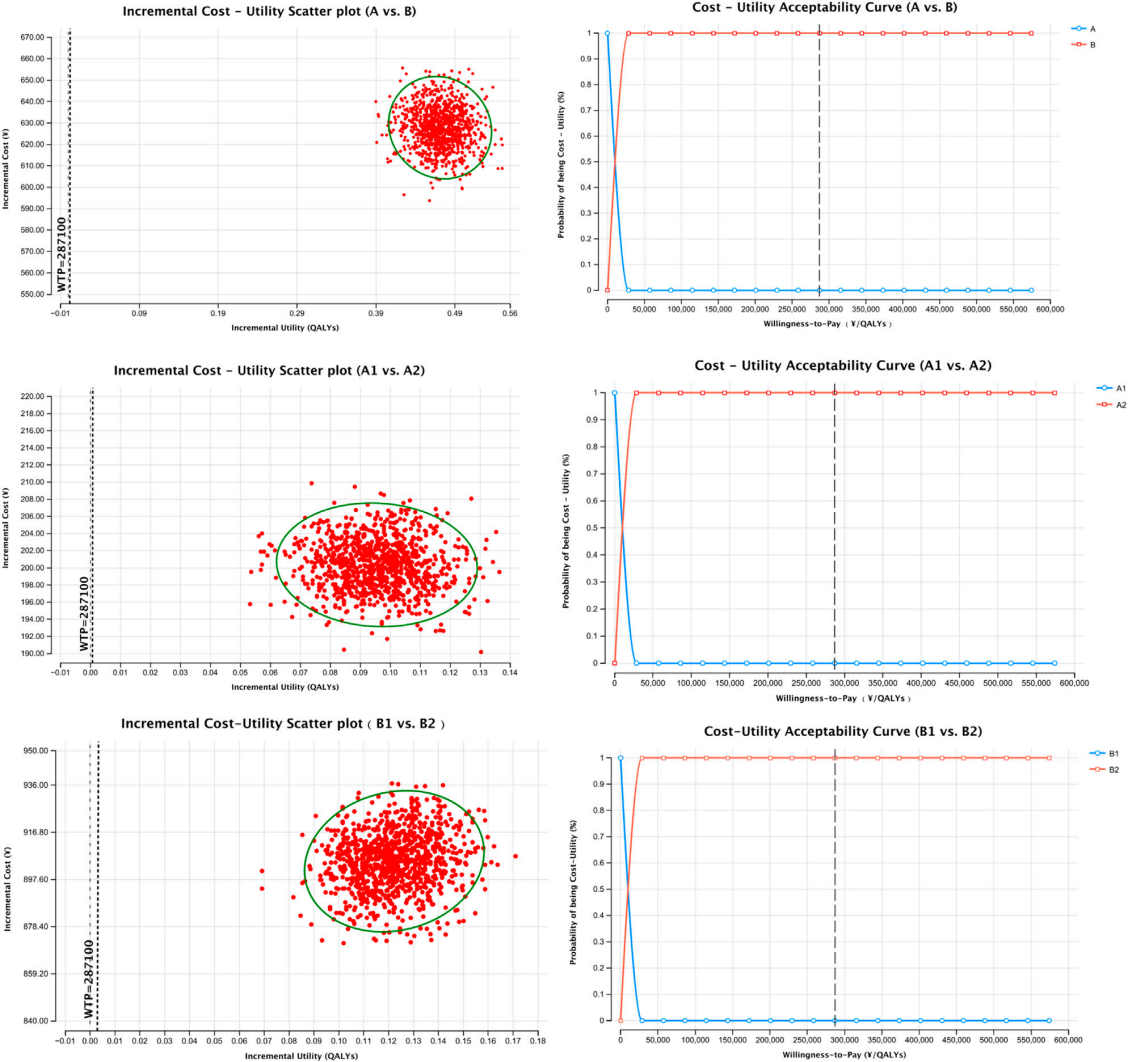
Sensitivity analysis of ICS cost-utility.

Cost-utility scatter plots based on 1,000 Monte Carlo simulations and cost-effectiveness acceptability curves (CEACs) are presented in Figures 1, 2. Regardless of whether total pharmacotherapy costs or ICS-specific costs were used as the input, the results remained robust and consistent, with most simulations falling within the 95% confidence interval. Sensitivity analyses corroborated the base-case findings, further supporting that triple ICS therapy is the most cost-effective, followed by dual therapy, with BF outperforming SF in single-agent scenarios. Parameters of the decision tree model are provided in Table 9, and detailed cost-utility outcomes are summarized in Table 10.

**TABLE 9:** Decision tree model parameters.

Variable	A			B		
	A1	A2	Total	B1	B2	Total
ICS cost (¥)						
Hospital visits	132.26	197.40	180.03	145.31	143.68	144.86
Outpatient visits for onset	44.09	141.00	106.80	159.81	327.08	215.56
Outpatient visits for no-onset	247.99	502.23	432.09	857.25	1881.11	1139.70
Total medication cost (¥)						
Hospital visits	1989.60	1979.70	1982.34	1746.83	1839.83	1772.49
Outpatient visits for onset	242.69	573.70	456.87	627.79	1002.77	752.78
Outpatient visits for no-onset	1652.40	1124.17	1269.89	3066.38	5923.50	3854.55
P (0.0000–1.0000)						
Hospital visits	0.1739	0.1474	0.1525	0.0640	0.0394	0.0548
Outpatient visits for onset	0.1739	0.1789	0.1780	0.1366	0.1232	0.1316
Outpatient visits for no-onset	0.6522	0.6737	0.6695	0.7994	0.8374	0.8135
Utility (QALYs)						
Hospital visits	11.6460	11.6460	11.6460	11.6460	11.6460	11.6460
Outpatient visits for onset	13.8060	13.8060	13.8060	13.8060	13.8060	13.8060
Outpatient visits for no-onset	15.6060	15.6060	15.6060	15.6060	15.6060	15.6060

**TABLE 10:** Cost-utility analysis results.

Variable	A			B		
	A1	A2	Total	B1	B2	Total
ICS cost- utility analysis						
ICS cost (¥)	192.41	392.67	335.75	716.42	1621.20	963.47
Incremental cost (¥)	-	200.27	-	-	904.78	627.72
Utility (QALYs)	14.60	14.70	14.68	15.11	15.23	15.15
Incremental Utility (QALYs)	-	0.10	-	-	0.12	0.47
Incremental cost/Incremental Utility	-	2087.43	-	-	7444.57	1335.52
Medication cost- utility analysis						
Medication cost (¥)	1465.89	1151.80	1233.82	2648.82	5156.37	3332.05
Incremental cost (¥)	314.09	-	-	-	2507.55	2098.23
Utility (QALYs)	14.60	14.70	14.68	15.11	15.23	15.15
Incremental Utility (QALYs)	−0.10	-	-	-	0.12	0.47
Incremental cost/Incremental Utility	−3273.86	-	-	-	20632.18	4464.17

## 4 Discussion

Asthma is the most prevalent chronic respiratory disease among children, characterized by a prolonged disease course that can significantly impact physical health, quality of life, and academic performance. It also imposes a substantial economic burden on affected families and places considerable strain on public healthcare systems (Global Initiative for Asthma, 2025; Asher et al., 2021; Asher and Pearce, 2014). In China, where healthcare resources are unevenly distributed, treatment decisions for pediatric asthma must consider not only clinical efficacy but also cost-effectiveness, in order to support rational allocation of public health resources. The stepwise approach recommended in the Global Initiative for Asthma (GINA) guidelines (Global Initiative for Asthma, 2025), as well as national and international guidelines (Gaillard et al., 2021; Editorial Board et al., 2020), emphasizes individualized asthma management through periodic assessment, treatment adjustment, and regular follow-up. Inhaled corticosteroids (ICS), either as monotherapy or in combination with long-acting β_2_-agonists (LABA), remain the cornerstone of maintenance therapy for pediatric asthma. However, the optimal pharmacoeconomic strategy for ICS use in children remains under debate, particularly given differences in age, disease severity, and healthcare settings.

Recent high-level evidence from a Cochrane systematic review has shown that, in patients with mild asthma (including adolescents and adults), as-needed low-dose ICS-formoterol is more effective in reducing exacerbations and the need for systemic corticosteroids compared to short-acting β_2_-agonist (SABA) monotherapy (Crossingham et al., 2021). In addition, maintenance and reliever therapy using very-low-dose ICS-formoterol has demonstrated equivalent symptom control while further lowering the risk of disease deterioration, relative to fixed-dose ICS or ICS-LABA regimens. In the context of chronic obstructive pulmonary disease (COPD), a randomized controlled trial by Muiser S compared as-needed budesonide-formoterol with fixed-dose fluticasone-salmeterol and found no significant difference in clinical efficacy, despite lower ICS exposure in the as-needed group (Muiser et al., 2023). While such findings offer valuable insights, most existing studies focus on adult populations or children older than 6 years. Data specific to preschool children (0–6 years)—a population with unique pathophysiological and pharmacokinetic considerations—remain limited. A meta-analysis by Zhou XJ suggested that fluticasone-salmeterol may provide superior efficacy compared to montelukast in children and adolescents aged 4–18 years (Zhou et al., 2021). Furthermore, a randomized, double-blind, multicenter study by Yoshihara S conducted in children under 4 years of age found that while combination therapy with fluticasone-salmeterol did not significantly outperform fluticasone monotherapy in terms of primary clinical outcomes, it was associated with better asthma control scores on the Japanese Pediatric Asthma Control (JPAC) scale (Yoshihara et al., 2019).

From a pharmacoeconomic perspective, existing research has predominantly focused on cost-effectiveness comparisons between individual inhaled medications. However, systematic evaluations of combination strategies involving multiple inhaled agents administered throughout the course of asthma treatment remain limited. This study is the first to comprehensively assess, from a longitudinal treatment perspective, the clinical effectiveness and pharmacoeconomic value of ICS-LABA monotherapy *versus* ICS-LABA and/or ICS-based combination regimens in children aged 0–18 years with asthma. The findings aim to provide evidence to inform individualized treatment optimization and support rational allocation of healthcare resources in pediatric asthma management.

A total of 59 children diagnosed with asthma were included in the study and categorized into two primary groups based on their inhaled medication regimens: the single-inhaler therapy group (Group A) and the multiple-inhaler regimens group (Group B). Group A was further divided into subgroups A1 (fluticasone, SF) and A2 (budesonide, BF), while Group B included B1 (dual-medicine combinations: SF + FP, SF + BF, or FP + BF) and B2 (triple-medicine combination: SF + BF + FP). In terms of demographic characteristics, male children accounted for a significantly higher proportion of the study population (72.88%) compared to female children (27.12%) (P < 0.05), particularly in subgroups B1 and B2. This finding is consistent with some studies indicating that the prevalence and severity of asthma exhibit gender differences at different ages: asthma is more prevalent and severe in males than in females before puberty, whereas it is more common in women (Chowdhury et al., 2021; Jenkins et al., 2022). Age distribution was consistent with the approved indications for inhaled therapy, with children aged 4–7 years comprising the largest group (66.10%), followed by those aged 8–14 years (22.03%) and 1–3 years (10.17%). Only one child was older than 14 years, and none were younger than 1 year. Notably, the vast majority of patients (86.44%) had a history of allergic conditions, such as allergic rhinitis, eczema, or familial asthma, in line with GINA guidelines, which identify such history as a major risk factor for pediatric asthma (Global Initiative for Asthma, 2025). No significant differences in infection rates during hospitalization were found across groups or subgroups, suggesting that acute infections were not the main precipitating factor for asthma in this cohort. Interestingly, seasonal analysis revealed that the A2 subgroup had significantly more summer visits compared to A1, although no seasonal differences were noted in other comparisons. This finding contrasts with previous studies that identified autumn as the peak season for asthma exacerbations in children in the Northern Hemisphere (Pike et al., 2018; Li et al., 2025). The discrepancy may be attributable to the relatively small sample size and highlights the need for larger studies to explore seasonal patterns in asthma exacerbation more robustly.

Clinical efficacy was assessed using the number of asthma-related visits and the maximum duration of symptom-free periods. Group B had a significantly higher total number of clinical visits than Group A (P < 0.05), with B2 having notably more visits than B1. Additionally, outpatient follow-up durations were significantly longer in Group B, indicating that patients in the multiple-inhaler regimens group tended to have more complex or severe disease presentations. In terms of symptom control, a higher proportion of patients in Group B achieved sustained remission across all time thresholds (≥3, ≥6, ≥12, and ≥24 months) compared to Group A, suggesting superior asthma control with multiple-inhaler regimens therapy. Despite the clinical benefits, asthma is a chronic disease that requires long-term pharmacologic management, which can place a significant financial burden on families and healthcare systems. Therefore, evaluating the cost-effectiveness and cost-utility of different inhalation regimens is essential to ensure that effective asthma control is achieved in an economically sustainable manner. The present study provides important real-world evidence supporting the pharmacoeconomic value of individualized, severity-based treatment strategies for pediatric asthma.

Medical costs are generally categorized into direct, indirect, and intangible costs. Direct costs encompass actual expenditures associated with disease prevention, diagnosis, and treatment, including outpatient consultation fees, pulmonary function tests, and medication costs. Indirect costs refer to productivity losses resulting from caregiver absenteeism, disability, or premature death. Intangible costs include psychological distress and social impact, which are inherently difficult to quantify in monetary terms. Given the pediatric focus of this study and the heterogeneity in family socioeconomic status, it was not feasible to consistently capture indirect and intangible costs. Consequently, only direct medical costs were included in the pharmacoeconomic evaluation. These were further stratified into non-medication costs and medication-related costs, the latter comprising expenditures on inhaled corticosteroids (ICS), traditional Chinese medicines (TCMs), and other pharmacologic agents. To reflect the real-world diversity in healthcare utilization across different disease states—including asymptomatic periods, outpatient management of acute exacerbations, and inpatient treatment—we conducted a comprehensive pharmacoeconomic evaluation from multiple perspectives.

A comparative analysis of direct treatment costs revealed no significant differences between groups in terms of total hospitalization cost, non-medication costs, medication costs, ICS costs, or other medication costs during inpatient treatment for acute exacerbations. The duration of hospital stays was also comparable across groups, with a mean length of stay under 10 days, suggesting a high level of standardization in inpatient asthma management. No significant differences were found in average daily hospitalization costs between Groups A and B or among their respective subgroups, with the exception of Group A2, which had a significantly higher average daily ICS cost than Group A1 (P < 0.05), likely due to the higher unit price of budesonide (BF) compared to fluticasone (SF). These findings reinforce the standardized nature of hospital-based treatment protocols for pediatric asthma.

In outpatient care (encompassing both acute exacerbation and asymptomatic phases), Group B exhibited higher medication, ICS, and other medication costs than Group A, while non-medication and TCM costs were not significantly different between the two groups. Further subgroup analysis revealed no significant cost differences between A1 and A2 or between B1 and B2. During the asymptomatic phase, all categories of outpatient costs were generally higher in Group B than in Group A, which may be attributed to greater disease complexity, longer treatment durations, and more frequent medication adjustments. This pattern aligns with GINA’s recommended “assessment–treatment adjustment–monitoring” strategy.

After standardizing outpatient costs on a monthly basis, no significant differences were observed between Groups A and B in average monthly total cost, non-medication cost, medication cost, TCM cost, or other medication cost, with the exception of monthly ICS cost, which was significantly higher in Group A (P < 0.05). This suggests that while total outpatient costs were higher in Group B due to prolonged treatment duration, the average monthly burden was comparable to that of monotherapy. Among monotherapy subgroups, Group A2 (BF) had significantly higher average monthly ICS costs than Group A1 (SF) (P < 0.05), reflecting the higher unit cost of BF. Conversely, Group A1 incurred significantly higher average monthly medication and TCM costs than Group A2 (P < 0.05, respectively), suggesting that when ICS doses are lower, additional adjunctive therapies—particularly TCMs—may be used to enhance treatment efficacy.

Interestingly, average monthly outpatient costs during the asymptomatic period were generally higher than during acute exacerbations, with the exception of TCM costs in the SF monotherapy group, which were higher during acute episodes. This may be due to the more frequent use of TCMs in combination with SF during acute exacerbations to improve clinical outcomes, highlighting a potential role for TCMs in the acute management of pediatric asthma that warrants further investigation.

In summary, the comparative analysis of direct medical costs across treatment regimens demonstrated that although combination therapy involving multiple ICS-LABA agents was associated with higher total medication and ICS costs compared to single-inhaler therapy, these differences diminished when adjusted for monthly expenditure. Among ingle-inhaler therapies, BF was more expensive than SF due to its higher unit price. Cost differences between dual and triple ICS-LABA combination therapies were relatively minor. Furthermore, hospitalization costs during acute exacerbations did not differ significantly between groups, reinforcing the consistency and standardization of inpatient care for pediatric asthma.

Cost-effectiveness analysis (CEA) is one of the most widely used approaches in pharmacoeconomic research. Its core principle is to compare the differences in costs required to achieve a unit of therapeutic effect among different treatment regimens. A commonly employed metric is the cost-effectiveness ratio (C/E), defined as C/E = cost/effect. Generally, a lower C/E value indicates a lower cost per unit of therapeutic benefit and thus reflects greater economic efficiency. However, because the C/E ratio is a relative measure, it is often supplemented with the incremental cost-effectiveness ratio (ICER) when comparing two regimens with differing costs and effectiveness. ICER is calculated as ICER = ΔC/ΔE, representing the additional cost required to gain one extra unit of effect. A positive ICER greater than the C/E of the lower-cost option may indicate poor economic favorability, while a negative ICER suggests that the new intervention is both more costly and less effective—thereby considered dominated and economically suboptimal.

In this study, both C/E and ICER metrics were used to evaluate the pharmacoeconomic value of different ICS-LABA regimens in pediatric asthma. Treatment effectiveness was operationalized as the proportion of patients achieving specific durations of the longest symptom-free period: E_3_ (≥3 months), E_6_ (≥6 months), E_12_ (≥12 months), and E_24_ (≥24 months). Cost indicators included total medication costs (C_M_) and ICS-specific costs (C_I_). The results showed that Group A (single-inhaler therapy: SF or BF) incurred significantly lower total medication costs than Group B (multiple-inhaler regimens: dual or triple ICS-LABA combinations) (P < 0.05). At the E_3_ timepoint, Group A exhibited a lower C/E ratio than Group B, indicating superior short-term cost-effectiveness. However, from E_6_ to E_24_, Group B demonstrated more favorable ICER values, suggesting improved long-term economic efficiency for combination therapy. In subgroup analysis, although no significant difference in total medication costs was found between Groups A1 (SF) and A2 (BF), A1 consistently showed better short-term cost-effectiveness. Within Group B, B1 (two inhalers, SF + FP, SF + BF, or FP + BF) incurred significantly lower total treatment costs than B2 (three inhalers, SF + BF + FP) and achieved more favorable C/E ratios across all timepoints except E_24_, where B2 demonstrated a slight advantage.

Analysis of ICS-specific costs revealed a similar trend. Group A had significantly lower ICS-related costs than Group B (P < 0.05). Group A maintained superior C/E ratios at E_3_, E_6_, and E_12_, with only a marginal disadvantage at E_24_. ICER analysis further confirmed that combination therapy (Group B) only offered economic advantages in complex cases requiring long-term management. At the subgroup level, Group A1 outperformed A2 in both ICS costs and C/E ratios. Moreover, Group A2 exhibited negative ICER values in several scenarios, suggesting that it was a dominated option in terms of cost-effectiveness. Group B1 generally outperformed B2 across most timepoints, with B2 showing a slight cost advantage only at E_24_.

In summary, analysis of both total medication costs and ICS-specific expenditures indicates that for mild disease and shorter treatment durations, monotherapy—especially with SF—offers superior economic efficiency. In contrast, combination therapy provides greater cost-effectiveness for more complex cases requiring prolonged treatment. Among combination regimens, triple-medicine therapy demonstrated slightly better control of total medication costs, while dual-medicine therapy was more favorable in terms of ICS-specific cost-effectiveness. These findings support the implementation of a stratified and individualized treatment strategy based on disease severity and expected treatment duration, with the goal of optimizing healthcare resource utilization while ensuring sustained clinical effectiveness.

In addition to cost-effectiveness analysis, this study also employed cost-utility analysis (CUA) to further evaluate the economic viability of different ICS-LABA treatment strategies for pediatric asthma. A decision tree model was constructed using TreeAge Pro 2022 software, incorporating a willingness-to-pay (WTP) threshold of three times China’s *per capita* GDP in 2024 (287,100 CNY/QALY). Health utility values were assigned to different disease states, including asymptomatic periods, acute exacerbation managed in outpatient settings, and hospitalization. Based on these utility weights, quality-adjusted life years (QALYs) and incremental cost-utility ratios (ICURs) were calculated for each treatment strategy.

The results demonstrated that, whether total medication costs or ICS-specific costs were considered, Group B (multiple-inhaler regimens: dual or triple ICS-LABA combinations) had a more favorable ICUR compared to Group A (single-inhaler therapy: SF or BF), with values of 4,464.17 CNY/QALY and 1,335.52 CNY/QALY, respectively—both well below the WTP threshold. These findings indicate that therapy with multiple inhalers is more cost-effective across the entire course of treatment. In subgroup comparisons, triple therapy (B2) yielded greater QALY gains than dual therapy (B1), with an ICUR of 20,632.18 CNY/QALY, indicating its greater economic benefit despite higher costs. Among single-inhaler therapies, although BF (A2) was associated with slightly higher costs than SF (A1), it achieved a more substantial utility gain, resulting in an ICUR of 2,087.43 CNY/QALY, also below the WTP threshold. Notably, despite the lower cost of SF, its reduced utility led to a negative ICUR, suggesting that BF therapy is more cost-effective.

To test the robustness of the model, sensitivity analysis was conducted using Monte Carlo simulation, with results visualized through cost-utility scatter plots and acceptability curves. The majority of simulations fell within the 95% confidence interval, supporting the model’s reliability and the consistency of the cost-utility outcomes.

Taken together, these results suggest that for children with more severe or persistent asthma, triple or dual ICS-LABA therapy provides greater cost-utility compared to single-inhaler therapy. Among single-agent regimens, BF appears to be more cost-effective than SF. These findings align with the cost-effectiveness results and further substantiate the value of stratified treatment approaches in pediatric asthma.

This study has several limitations. First, the diagnosis of asthma was based on the 2016 Guidelines of the Chinese Pediatric Society Respiratory Group, which were the officially recognized criteria in China during the study period. Although these guidelines ensured consistency with local practice, more recent international standards, such as GINA 2025 (Global Initiative for Asthma, 2025) and BTS/NICE/SIGN 2024 (British Thoracic Society BTSNational Institute for Health and Care Excellence NICEScottish Intercollegiate Guidelines Network SIGN, 2025), provide updated diagnostic and management criteria. Future studies incorporating these international guidelines would enhance the generalizability and comparability of the findings. Second, it only included children who were hospitalized in the respiratory department of our hospital and received inhalation therapy from January to December 2023, resulting in a relatively small sample size. This may affect the representativeness and generalizability of the results. Future studies should aim to expand the sample size and conduct multi-center research to enhance the external validity of the findings. Third, there was an imbalance in sample sizes between treatment groups, with some groups containing nearly three times more patients than others. This uneven distribution may have introduced potential bias in the statistical analyses. Given the retrospective and single-center nature of the study, patient allocation across groups was not randomized. Fourth, this study only analyzed specific formulations of certain inhaled medications, including salmeterol-fluticasone inhalation powder (50 μg/100 μg/puff), budesonide-formoterol inhalation powder (80 μg/4.5 μg/puff), and fluticasone propionate metered-dose inhaler (125 μg/puff), without covering other formulations or dosages, which could affect the economic evaluation in different clinical scenarios. Fifth, as a retrospective study, there may be missing or incomplete data on patients’ medical histories and relevant evaluation indicators. Sixth, only direct medical costs were included, without accounting for indirect costs (such as caregiver absenteeism and transportation expenses) or intangible costs (such as family burden and quality of life impact), potentially underestimating the true economic burden of asthma treatment. Lastly, due to the age-stratified management strategy for inhaled medications in our hospital, there were variations in age distribution among the treatment groups, which may have influenced the accuracy of cost-effectiveness and cost-utility analyses. Additional, we observed a higher proportion of male children in our cohort, particularly in combination therapy subgroups. Although our analysis did not find a significant association between gender and the frequency of exacerbations, gender distribution may act as a potential confounding factor, representing another limitation of the study. Future studies should focus on prospective, multi-center research including a wider age range, particularly children under 6 years of age, and consider stratifying analyses by gender to comprehensively evaluate the safety, efficacy, and economic outcomes associated with ICS-LABA therapies in pediatric asthma treatment.

In summary, addressing these limitations in future research will enhance the robustness of findings and contribute to stronger evidence for optimizing clinical treatment strategies and informing relevant drug policies in the management of pediatric asthma.

## 5 Conclusion

This study systematically evaluated the economic efficiency of various ICS-LABA inhalation strategies in the treatment of hospitalized pediatric asthma patients using both cost-effectiveness and cost-utility analyses. The findings indicate that in managing complex or prolonged asthma cases, multiple inhaler regimens are more cost-effective than single-inhaler therapy, with triple therapy offering advantages in terms of total medication costs, and dual therapy showing better ICS-specific cost-effectiveness. For milder conditions and shorter treatment durations, single-inhaler therapy remains economically favorable, with BF outperforming SF in terms of cost-utility.

Both ICER and ICUR analyses confirmed that although multiple-inhaler regimens incur higher costs, these increases are offset by meaningful gains in treatment effectiveness, falling well within acceptable cost thresholds. Sensitivity analysis further demonstrated the robustness of the pharmacoeconomic model and the reliability of the conclusions.

Overall, this research provides valuable evidence to support individualized, economically sound treatment planning in pediatric asthma and offers critical insights into the optimal allocation of medical resources and cost-effective drug utilization strategies in clinical practice.

## Data Availability

The original contributions presented in the study are included in the article/supplementary material, further inquiries can be directed to the corresponding author.
